# Temporality, lived time and psychopathology of everyday life

**DOI:** 10.1192/j.eurpsy.2021.2035

**Published:** 2021-08-13

**Authors:** M. Carneiro, S. Nascimento, T. Coelho Rocha, J. Cunha

**Affiliations:** 1 Serviço Psiquiatria E Saúde Mental, Centro Hospitalar Barreiro Montijo, Barreiro, Portugal; 2 Psychiatry, Centro hospitalar psiquiátrico de lisboa, Lisboa, Portugal

**Keywords:** philosophy, temporality, phenomenology, psychopathology

## Abstract

**Introduction:**

Since Ancient Times, Man has tried to analyze the passage of time, looking for repetitions, relating them to space to build a notion of a mechanical and chronological time. The idea and problem of time play a central role in both modern philosophy and psychiatry. Many authors contributed to the notion of “lived time” and placed the focus on how time is lived and perceived by the individual. Even though the notion of “time assimilated in space” has an important role in psychiatric nosology, the “lived time” has a psychopathological impact and is a field of study and debate.

**Objectives:**

This work aims to acknowledge the relevance of the experience of temporal structures (past, present and future) and how they relate to psychopathology.

**Methods:**

We did a non-systematic literature revision in the main databases.

**Results:**

Phenomenological psychopathology has been profoundly interested in the philosophical discussions on the nature of time and its relation with the subject’s experience and condition. For instance, the melancholic experience, the maniac experience and the schizophrenic experience constitute changes in how time structures are perceived and lived by the individual.

**Conclusions:**

Temporality has drawn attention to researchers from many different areas of study, having as of this day many approaches possible. It is important to know those contributions and conceptualizations in order to improve as a clinician.

**Disclosure:**

No significant relationships.

